# Effect of a Diet Supplemented with Marine Omega-3 Fatty Acids on Inflammatory Markers in Subjects with Obesity: A Randomized Active Placebo-Controlled Trial

**DOI:** 10.3390/healthcare13020103

**Published:** 2025-01-08

**Authors:** Joel Torres-Vanegas, Roberto Rodríguez-Echevarría, Wendy Campos-Pérez, Sarai Citlalic Rodríguez-Reyes, Samantha Desireé Reyes-Pérez, Mariana Pérez-Robles, Erika Martínez-López

**Affiliations:** 1 Doctorado en Ciencias de la Nutrición Traslacional, Centro Universitario de Ciencias de la Salud, Universidad de Guadalajara, Guadalajara 44100, JA, Mexico; 2 Instituto de Nutrigenética y Nutrigenómica Traslacional, Centro Universitario de Ciencias de la Salud, Universidad de Guadalajara, Guadalajara 44100, JA, Mexico; 3 Departamento de Biología Molecular y Genómica, Centro Universitario de Ciencias de la Salud, Universidad de Guadalajara, Guadalajara 44100, JA, Mexico; 4 Doctorado en Ciencias en Biología Molecular en Medicina, Centro Universitario de Ciencias de la Salud, Universidad de Guadalajara, Guadalajara 44100, JA, Mexico

**Keywords:** diet, obesity, omega-3, low-grade inflammation, specialized pro-resolving mediators, inflammatory markers

## Abstract

Background/Objectives: Obesity is associated with chronic low-grade inflammation. Polyunsaturated fatty acids (PUFAs) such as omega-3 (n-3), are essential in anti-inflammatory processes. Therefore, the aim of this study was to evaluate the effect of a dietary intervention along with supplementation of 1.8 g of marine n-3 PUFAs on anthropometric, biochemical, and inflammatory markers in adults. Methods: An 8-week double-blind randomized clinical trial was conducted with a diet (200 kcal/day reduction each 4 weeks based on the estimated basal caloric expenditure) containing a n-6/n-3 PUFA ratio ≤ 5:1, along with daily 1.8 g of marine n-3 supplementation (EPA and DHA) vs. active placebo 1.6 g (ALA). A total of 40 subjects were included in the study, 21 in the marine omega-3 group and 19 in the active placebo group. Inclusion criteria included subjects aged 25 to 50 years with obesity as determined by body mass index (BMI) and/or abdominal obesity according to ATP III criteria. Results: The marine omega-3 supplemented group had a better effect compared to the active placebo group, increasing Resolvin D1 [129.3 (−90.1–193.5) vs. −16.8 (−237.8–92.5) pg/mL, *p* = 0.041], IL-10 [1.4 (−0.7–4.6) vs. −2.0 (−5–0.05) pg/mL, *p* = 0.001], and decreasing IL-6 [−0.67 (2.72–−0.59) vs. 0.03 (−0.59–1.84) pg/mL, *p* = 0.015], and MCP-1 [−29.6 (−94.9–5.50) vs. 18.3 (−97.3–66.35) pg/mL, *p* = 0.040]. Conclusions: A diet supplemented with marine n-3 improves inflammatory markers by increasing systemic levels of Resolvin D1 and IL-10 and decreasing IL-6 and MCP-1.

## 1. Introduction

Obesity (OB) is characterized by an excessive accumulation of body fat resulting from an imbalance between daily energy intake and energy expenditure, leading to excess weight. This condition is a multifactorial disease that is influenced by various genetic, cultural, and social factors. Contributing factors include reduced physical activity, insomnia, endocrine disorders, medications, and imbalances in fat intake such as polyunsaturated fatty acids (PUFAs), omega-6 (n-6), and omega-3 (n-3) that are mainly related to metabolic disturbances commonly present in obesity [1,2]. Moreover, OB is associated with numerous comorbid and chronic medical conditions, including an elevated risk of type 2 diabetes mellitus, cardiovascular disease, hypertension, and dyslipidemia [3].

Even though the n-3 has been widely associated with better metabolic status, currently, a low intake of PUFAs, accompanied by an imbalance in the n-6/n-3 ratio, has been associated with many pro-inflammatory conditions and a worse metabolic health [1,2,4,5]. In Mexico, an n-6/n-3 ratio of 12:1 has been reported, whereas the recommended ratio suggests a consumption ratio of no more than 5:1 [6,7,8]. This imbalance is strongly related to overweight comorbidities and may contribute to the chronic low-grade inflammation [4,9].

On the other hand, the inflammatory response functions as a defense mechanism in the innate immune system to protect the host from harmful stimuli; however, its activity must cease once the threat has subsided. This self-limiting process is crucial for preserving homeostasis [10]. Nevertheless, the failure to achieve resolution, or continued exposure to environmental and biological factors that induce inflammatory response activation, can lead to a chronic inflammatory process. This results in the prolonged presence of immune cells such as lymphocytes, macrophages, and plasma cells in the adipose tissue, accompanied by the secretion of pro-inflammatory cytokines [11,12].

The prolonged existence of chronic low-grade inflammation triggers the infiltration of immune cells and the secretion of inflammatory cytokines within the tissue environment, potentially inhibiting glucose absorption and altering lipid metabolism [13]. Currently, attention has been focused on investigating the biological resolution of inflammation initiated by specific biochemical signals generated through the ingestion of n-3 PUFAs. These potent bioactive molecules are collectively known as specialized pro-resolving mediators (SPMs), which include resolvins (Rv), protectins (PD), and maresins (MaR) [14]. The beneficial effect of the marine n-3 PUFA supplementation on inflammatory markers has been studied [15]. In addition, studies have reported that the biological effect of n-3 is influenced by other factors including the BMI [16,17] and the dietary n-6/n-3 ratio [5] which suggests that both variables should be evaluated in future studies.

Therefore, it is important to highlight that one of the purposes of this study is to work with menus designed with an adequate n-6/n-3 PUFA ratio ≤ 5:1. The aim of this study was to evaluate the effect of this dietary intervention with 1.8 g of marine n-3 PUFAs on anthropometric, biochemical, and inflammatory markers in adults with obesity.

## 2. Materials and Methods

### 2.1. Study Design

A randomized, active placebo-controlled, double-blind parallel clinical trial with eight weeks of follow-up was carried out at the Instituto de Nutrigenética y Nutrigenómica Traslacional between October and December 2021. This trial was approved by Ethics Committees of the Centro Universitario de Ciencias de la Salud, Universidad de Guadalajara (CI-03221). Furthermore, this study was registered on ClinicalTrials.gov (NCT05068557) and conducted according to the Declaration of Helsinki (2013) [18]. Written informed consent was obtained from participants before screening and data collection.

### 2.2. Study Participants

**Figure 1:**
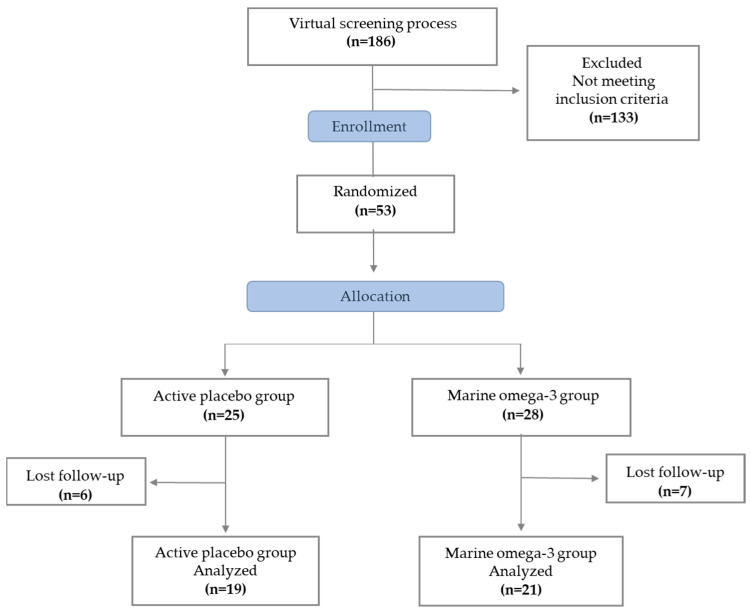
Flow diagram of subjects included in the study.

All participants were recruited through flyers and social media invitations. A virtual screening process was conducted using electronic forms to assess eligibility. Inclusion criteria included subjects aged 25–50 years with obesity, defined as a body mass index (BMI) of ≥30 kg/m^2^ obesity [19], and/or abdominal obesity as defined by ATP III criteria [20]. Subjects who were pregnant, lactating, taking either n-3, n-6, or n-9 supplements, using anti-inflammatory, lipid-lowering, hypoglycemic, antihypertensive medications, or who had immunological or neoplastic diseases, history of cholecystectomy, or fish or shellfish allergies or intolerances, were not included. Those with a current COVID-19 diagnosis or within the previous 30 days were excluded. A total of 40 Mexican adults with obesity were recruited (Figure 1).

### 2.3. Randomization

The supplement assignment was accomplished using the randomly permuted blocks method to balance the number of subjects assigned to each treatment group [21]. The procedure was conducted by a researcher who was not involved in the treatment or follow-up of the subjects and who was responsible for recording and controlling the double-blinded intervention.

### 2.4. Intervention

**Figure 2:**
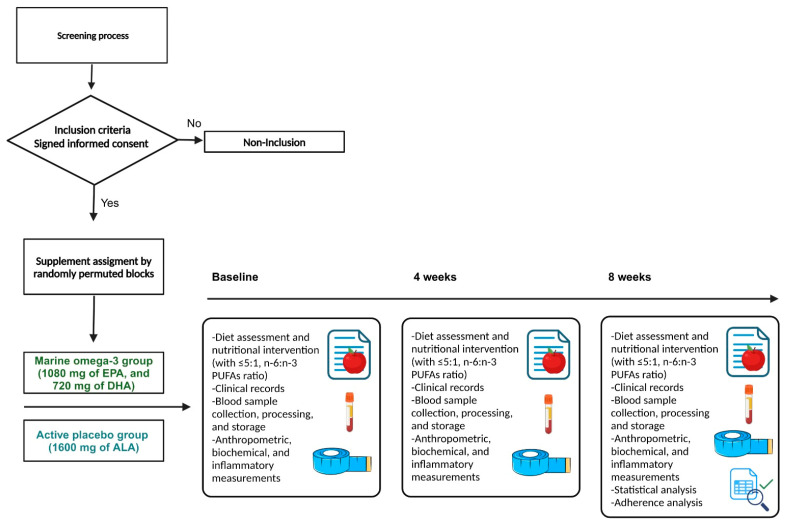
Intervention.

The intervention consisted of a diet, which was the same for both groups, and supplementation with either marine n-3 or an active placebo depending on the study group. All the participants underwent interviews with a nutritionist to obtain clinical records and sociodemographic information. Additionally, a 3-day dietary record was collected using food replicas (Nasco, Fort Atkinson, WI, USA) to assist in portion size estimation in accordance with the Sistema Mexicano de Alimentos Equivalentes [22] (Figure 2).

#### 2.4.1. Nutritional Intervention

The nutritional intervention consisted of a 200 kcal/day caloric restriction during the first 4 weeks, followed by an additional 200 kcal/day in the last 4 weeks, based on the basal estimated caloric expenditure using the Mifflin St. Jeor equation [23]. A recipe book was developed by nutritionists, which includes 20 days of 3 main meals and 2 snacks calculated to maintain a 5:1 ratio of n-6/n-3 PUFAs. Recipes were analyzed using Nutritionist Pro™ software (Version 8.1, Axxya Systems, Woodinville, WA, USA) according to NOM-043, NOM-037, and GPC guidelines [24,25,26].

#### 2.4.2. Marine Omega-3 and Active Placebo Intervention Groups

The administration of capsules in both groups consisted of 3 capsules per day, 1 capsule with each main meal. The marine n-3 supplementation consisted of 3 capsules per day containing 1080 mg of eicosapentaenoic acid (EPA) and 720 mg of docosahexaenoic acid (DHA), purchased from General Nutrition Center (GNC^®^, Pittsburgh, PA, USA). Double Strength Fish Oil No. 1001107013, lot number: 0384AT8916 (distributed by Maxiva, S.A. de C.V., Av. Vasconcelos No. 195, Col Santa Engracia, Postal Code 66267, San Pedro Garza García, Nuevo León) was used. Meanwhile, the active placebo group took 1600 mg of vegetable n-3 alpha-linolenic acid (ALA), purchased from Biocap Mexico^®^ No. C111197311, lot number: 1500113620 (chia and flaxseed oil capsules distributed by Biocaps de México S.A. de C.V. Cda. Filomeno González 1a, Ampliacion San Miguel Ajusco, Tlalpan, 14710 Ciudad de México, CDMX) which has been found to have no effects on inflammatory markers [27], representing an appropriate active placebo according to the literature [28,29]. Subjects were instructed to take 1 capsule with each main meal in both groups.

### 2.5. Outcome Measurements

#### 2.5.1. Dietetic Analyses

To assess the 3-day food records completed by participants (including 2 weekdays and 1 weekend day) at baseline and at week 8, the provided dietary information was analyzed using the Nutritionist Pro™ software (Version 8.1, Axxya Systems, Woodinville, WA, USA), where the average nutrient consumption was obtained. The dietary n-3 was calculated by adding the following PUFAs: alpha-linolenic acid (18:3), stearidonic acid (18:4), eicosapentaenoic acid (20:5), docosapentaenoic acid (22:5), docosahexaenoic acid (22:6). The n-6 PUFAs were calculated by summing the linoleic acid (18:2) and arachidonic acid (20:4). The n-6/n-3 ratio was calculated by dividing the n-6 value by the n-3 value in grams. In order to evaluate the n-6/n-3 PUFA ratio in the diet, the ratio ≤ 5:1 was considered as an ideal [7,8,30].

#### 2.5.2. Adherence Analyses

Regarding adherence, it was calculated taking into account the kcal consumed, the n-6/n-3 PUFA ratio, and the intake of capsules, where the kcal corresponds to 25%, the n-6/n-3 PUFA ratio to the other 25%, and the intake of capsules to the remaining 50%.

The 25% of the kcal and the 25% of the n-6/n-3 PUFA ratio were calculated based on their percentage of adequacy (i.e., a percentage of adequacy of 100% would correspond to 25% of adherence). On the other hand, to calculate the other 50% corresponding to the intake of capsules, the number of capsules ingested was counted based on the number of capsules that the patient returned at the end of the intervention. Finally, the total adherence was calculated by adding the percentage of adherence of the kcal, the n-6/n-3 PUFA ratio, and the intake of capsules.

#### 2.5.3. Anthropometric Measurements

Measurements were taken after 8 to 12 h of fasting, barefoot, and wearing light clothes. Height was determined by using a stadiometer with a precision of 1 mm (Rochester Clinical Research, Inc., New York, NY, USA) with the head in Frankfurt position, arms along the body, and shoulders, buttocks, and heels touching the device. Waist circumference (WC) was measured in the narrowest diameter between the last rib and the iliac crest according to the ISAK method using a Lufkin Executive^®^ thin line 2 mm measuring tape (New Brighton, MN, USA). Abdominal obesity was defined as WC ≥ 88 cm in women and ≥102 cm in men according to ATP-III [19]. Tetrapolar body electrical bioimpedance was used to assess body fat percentage (BF%) (InBody 570, Biospace Co., Seoul, Republic of Korea). In addition, BMI was calculated as weight in kilograms divided by height in meters squared (kg/m^2^).

#### 2.5.4. Biochemical Measurements

A peripheral blood sample was collected through the vacutainer system after 8 to 12 h of fasting, then centrifuged at 4 °C for 15 min at 3500 RPM to obtain serum and plasma, and finally stored at −80 °C for future analyses. Serum glucose, triglycerides (TG), total cholesterol (TC), and high-density lipoprotein cholesterol (HDL-c) were determined by dry chemistry using a Vitros 350 Analyzer (Ortho-Clinical Diagnostics, Johnson & Johnson Services Inc., Rochester, NY, USA). Very low-density lipoprotein cholesterol (VLDL-c) was estimated by dividing total triglycerides by 5. Low-density lipoprotein cholesterol (LDL-c) was calculated using the Friedewald formula if TG levels were less than 400 mg/dL [31]. Insulin was determined by a chemiluminescent immunoassay using a Liaison^®^ (DiaSorin S.p.A., Saluggia (VC), Italy), and insulin resistance was calculated using the homeostasis model assessment (HOMA-IR = fasting insulin (μIU/mL) × fasting glucose (mg/dL)/405] [32].

#### 2.5.5. Inflammatory Parameters

The cytokines TNF-α (Catalog A35601), IL-6 (Catalog A35573), MCP-1 (Catalog A35598), and IL-10 (Catalog A35590) were determined in serum samples using ProQuantum High Sensibility Immunoassay kits (Invitrogen by Thermo Fisher Scientific, Carlsbad, CA, USA). High-sensitivity C-reactive protein (hs-CRP) was measured by immunofluorescence using the Getein 1100 equipment, and plasma Resolvin D1 (RvD1) was quantified by competitive enzyme-linked immunosorbent assay (Cayman Chemical, Ann Arbor, MI, USA).

### 2.6. Statistical Analysis

The sample size was calculated using the OpenEpi v3 software with the mean difference formula for clinical trials. Reference values were obtained from changes in RvD1 levels reported in subjects with obesity receiving n-3 supplementation [33]. To achieve a statistical power of 80% and an alpha of 0.05%, a sample size of 19 participants in each study group was required.

To evaluate the normality distribution of the variables, the Shapiro–Wilk test was used. Normal quantitative variables were expressed as mean ± standard deviation and those with non-normal distribution as median and interquartile range (IQR). Qualitative variables were expressed as frequencies and percentages. For comparative analysis of normal variables between two independent groups or related samples, Student’s *t*-test was used, while for non-normal analyses, the Mann–Whitney U test or Wilcoxon test was used.

Delta (∆) was calculated to analyze the differences in the means of the groups through the intervention (∆ = final mean value − baseline mean value). Repeated measures analysis of variance (ANOVA) or Friedman tests were conducted, adjusted by biological sex as a covariate. To evaluate changes in qualitative variables in the study groups, the McNemar test was used. A *p*-value < 0.05 was considered statistically significant. Data collected during the study were first added to an Excel document, then all statistical analyses were carried out using the SPSS v25.0 software (IBM Corp., Armonk, NY, USA).

## 3. Results

A total of 40 subjects were included in the study, with 21 in the marine omega-3 group (38% (n = 8) females and 62% (n = 13) males) and 19 in the active placebo group (42% (n = 8) females and 58 % (n = 11) males), *p* = 0.894. At baseline, there were no statistically significant differences in the nutritional variables between the study groups (Supplementary Material, Table S1).

### 3.1. Characteristics of Diet and Adherence

The analysis of dietary variables is presented in Table 1 (baseline vs. 8 weeks post-intervention). The protein intake per kilogram of body weight at the final time point was 0.85 g/kg for the active placebo group and 0.91 g/kg for the marine omega-3 group. A significant increase was observed in n-3 PUFA intake in both groups. In contrast, in the marine omega-3 group, the intake of n-6 PUFAs significantly decreased compared to baseline consumption. Nonetheless, it is important to highlight that the n-6/n-3 ratio intake improved significantly in both groups, achieving an ideal proportion.

In the active placebo group, the total ALA intake per day (diet + capsules) was 1334 mg, whereas in the marine omega-3 group, it was 233 mg. Additionally, the intake of marine n-3 (diet + capsules) in the active placebo group was 0.452 mg, while in the marine omega-3 group, it was 1586 mg.

After adding the results regarding the percentage of adequacy of kcal consumption (25%), intake of PUFA n-6/n-3 ratio (25%), and the capsules ingested (50%), it was found that the active placebo group had an adherence percentage of 70 ± 18.2% and the marine omega-3 group of 77 ± 25.2% with no statistically significant differences (*p* = 0.207).

### 3.2. Anthropometric and Biochemical Measurements

In Table 2, the results of the anthropometric parameters throughout the intervention (baseline, 4 weeks, and 8 weeks) are presented. After intervention, improvements in weight, BMI, TG, and VLDL-c were observed in both groups. Moreover, in the omega-3 group, HDL-c, body fat percentage, and WC improved significantly in men [34]. However, when comparing intergroup differences, no statistical differences were observed.

Furthermore, the prevalence of abdominal obesity was significantly reduced by 35% in the omega-3 group (*p* = 0.016), compared to a 5.6% reduction in the active placebo group (Table 3). Based on the aforementioned, weight loss by gender was analyzed, and it was found that men in the marine omega-3 group had a greater weight loss of −4.2 kg (*p* = 0.007) compared to the active placebo group with a weight loss of −2.2 kg (*p* = 0.115).

Regarding fasting insulin and the HOMA index, an improvement was observed in both groups; however, no differences were found between groups. In the active placebo group, the basal insulin was 19.2 ± 9.3 μIU/mL vs. 15.5 ± 8.2 μIU/mL final (*p* = 0.040). Likewise, in the marine omega-3 group, a significant decrease in insulin was observed (20.0 ± 11.4 vs. 14.8 ± 11.4 μIU/mL, *p* = 0.025). On the other hand, the basal HOMA index in the marine omega-3 group was 4.8 ± 2.8 vs. 3.5 ± 2.4 final (*p* = 0.076), and in the active placebo group, it was 4.4 ± 2.1 vs. 3.62 ± 1.9 (*p* = 0.122).

### 3.3. Inflammatory Markers

After the intervention, TNF-α, IL-6, and MCP-1 decreased in the marine omega-3 group, while IL-10 and RvD1 increased. A decrease in TNF-α was also observed in the active placebo group, while better differences between IL-6, IL-10, and RvD1 changes were found in the marine omega-3 group (Table 4).

## 4. Discussion

Supplementation with n-3 PUFAs has been shown to play an important role in the treatment of obesity by having a beneficial effect on chronic low-grade inflammation. However, controversial results persist among different populations, particularly regarding the duration and consumption quantity [35]. The aim of this study was to evaluate the effect of a diet with an average ratio of 5:1 (n-6/n-3) supplemented with marine n-3 on anthropometric, biochemical, and inflammatory markers in adults with obesity [30].

Given the crucial role of subjects’ adherence in this type of interventional project, WHO’s recommendations for chronic disease treatments suggest achieving at least 50% adherence [36]. Therefore, the findings in this study indicate an adequate adherence, showing more than 70% in both groups. However, more motivational strategies should be implemented in further studies, along with satisfaction surveys, to improve adherence.

Regarding baseline analysis of the three dietary records, all subjects showed an imbalanced consumption of the n-6/n-3 PUFA ratio, which agrees with other studies in Mexican populations with obesity [6,37,38]. This imbalance has been associated with chronic low-grade inflammation, a higher percentage of adipose tissue, larger WC, and higher levels of triglycerides, glucose, and insulin [6,14].

In this study, both groups significantly increased their n-3 PUFA intake and achieved a dietary n-6/n-3 consumption ratio of <5:1 by the end of the intervention. However, it is noteworthy that the marine omega-3 group exhibited statistically lower n-6 dietary intake compared to the active placebo group. These findings could be related to a better metabolic profile.

The analysis of anthropometric and biochemical variables showed intragroup improvements in weight and BMI; however, in variables that must be analyzed by biological sex, it was found that body fat percentage and waist circumference were significantly better in men of the omega-3 group. These results could be attributed to the implementation of the same dietary recommendations in the provided recipe book, and the improvements in men in the omega-3 group could be due to the supplementation with better metabolic responses because as it has been reported that men lose weight more easily than women [39].

Nevertheless, the prevalence of abdominal obesity significantly decreased by 35% in the marine omega-3 group compared to 5.6% in the active placebo group. These findings are consistent with meta-analyses that found a relationship between n-3 intake from animal sources and better anthropometric measures [40,41]. However, the impact of these PUFAs on body composition has been controversial, with WC reported as the anthropometric variable related to obesity that has the most significant impact after n-3 supplementation [41].

Among the biochemical variables, some lipid profile components showed improvement, such as triglycerides and VLDL-c. This could be explained by a mechanism in which n-3 PUFAs act as ligands for peroxisome proliferator-activated receptor alpha (PPAR-α), a transcription factor that promotes the expression of genes involved in the β-oxidation of fatty acids [42,43], thereby decreasing VLDL-c levels. The VLDL-c is responsible for the endogenous transport of triglycerides from the liver to peripheral tissues, including visceral fat, mainly in the abdominal region [44]. On the other hand, no effects were observed on serum total cholesterol, findings consistent with a meta-analysis by Harrys et al., which showed that triglycerides and VLDL-c significantly decreased while total cholesterol levels were not altered after 7 to 10 weeks of n-3 PUFA supplementation [45]. Related to serum HDL-c, a decrease was observed at the end of the intervention in men of the marine omega-3 group. This could be related to other factors that affect HDL-c values, such as physical activity and genetic variability [46].

Regarding fasting insulin, improvements were observed during the intervention, independent of groups, which may be due to the same healthy dietary regimen and its favorable impact on insulin levels [47]. Furthermore, the HOMA index consistently displayed better changes in the marine omega-3 group throughout the intervention. It has been reported that the dietary n-6/n-3 PUFA ratio affects insulin resistance, where upon lowering the ratio, insulin resistance improves compared with higher ratios [48]. Moreover, activation of FFAR4 by n-3 fatty acids has been associated with enhancements in insulin sensitivity and glucose homeostasis [49,50]. In this study, we did not assess the activity of FFAR4; however, the evaluation of this pathway could be important in further research.

Nevertheless, the literature reports inconclusive effects of n-3 on these variables in humans [51], which may be due to the fact that glucose homeostasis is influenced by factors other than n-3 intake, such as lifestyle, physical activity, body fat percentage, among others [47,51,52]. Given this complexity, further clinical trials with longer periods are necessary, as our intervention lasted only 8 weeks and the activation of FFAR4 can be evaluated.

Other metabolic consequences of obesity associated with the imbalance of the n-6/n-3 PUFA ratio intake is the maintenance of chronic low-grade inflammation, primarily in adipose tissue [53,54]. Studies with in vitro and clinical trials have demonstrated that n-3 PUFAs, particularly EPA and DHA, decrease the number of resident macrophages in adipose tissue and crown-like structures characteristic of the M1 phenotype. Additionally, there is a decrease in levels of MCP-1, a chemotactic protein with a key role in the initiation, development, and perpetuation of chronic low-grade inflammation in obesity [55,56]. This is consistent with the findings of this study, where MCP-1 levels were lower in the marine omega-3 group at the end of the intervention.

Similarly, IL-6 concentrations were lower in the omega-3 group, both intra- and intergroup, at the end of the intervention, consistent with the study of Milutinivic et al., who evaluated the effect of n-3 supplementation (2.4 g/day) for 8 weeks on inflammatory markers, highlighting a statistically significant decrease in IL-6 [57]. Moreover, a meta-analysis published in 2021 by Wei Y. et al., including 31 studies between 2003 and 2019, aimed to study the effect of inflammatory markers and the n-6/n-3 PUFA ratio consumption in various inflammatory etiology diseases, emphasizing obesity and associated comorbidities. Omega-3 supplementation has been associated with the modulation of inflammation through the nuclear factor κB (NF-κB) pathway. Research indicates that n-3 fatty acids can influence markers of inflammation by inhibiting the production of pro-inflammatory cytokines and regulating the NF-κB signaling pathway [58]. As in this study, it was concluded that systemic levels of IL-6 and TNF-α were lower in subjects with lower n-6/n-3 consumption ratios as has been documented in the literature [59]. This could have a clinical impact on insulin sensitivity, as both groups improved their insulin serum concentrations. However, the HOMA index in the marine omega-3 group shows a favorable trend compared to the active placebo group. These findings are consistent with those reported by Xiao-fei Guo, who found a decrease in TNF-α associated with lower insulin concentrations [60]. However, it is important to note that the cytokines examined in this study were systemic ones; it is relevant to analyze in further studies the loco-regional cytokines involved in inflammation like TGF-β and IFN-γ, which have effects that include reducing regulatory T cells, diminishing their function, which appears to reduce adipose inflammation and suppress adipocyte metabolism, leading to better metabolic status [61].

Regarding RvD1, which belongs to the family of lipid pro-resolution inflammation mediators synthesized from DHA metabolism [14,62,63], it significantly increased compared to baseline in the marine omega-3 group along with IL-10. In this sense, it has been reported that RvD1 promotes the synthesis of IL-10 [64] which agrees with a study by Polus et al., which included 59 women with obesity who were provided with dietary guidelines and supplementation with 1.8 g/day of n-3 for 12 weeks. In proportion, they observed a 58% increase in RvD1 plasma levels and an increase in IL-10 compared to baseline in the n-3 supplemented group. Although our results are consistent, the higher increase in the Polus et al. study may be due to the longer intervention (12 vs. 8 weeks) [33]. This finding suggests that the chronicity of n-3 consumption favors the incorporation of these PUFAs into the cellular membrane phospholipids [7], and as a result, enhances the RvD1 synthesis, reducing the activation of the NF-κB-mediated cascade, which leads to a decrease in TNF-α, IL-6, and MCP-1, and an increase in IL-10 [62,65,66].

One of the strengths of this study was the calculation of dietary strategies considering the n-6/n-3 PUFA ratio intake. Additionally, the use of highly sensitive methodologies for measuring insulin and inflammatory markers was crucial due to the context of chronic low-grade inflammation, where concentrations of these markers were expected to be lower compared to acute inflammatory conditions.

One of the main limitations of this study is the small sample size aspect that could be improved in future research. Another significant limitation is the absence of a fatty acid analysis in erythrocytes; however, in a previous study conducted by our research group, the analysis was performed and it was found that supplementation with 1.5 g of marine n-3 PUFAs resulted in a greater incorporation percentage of these PUFAs into erythrocyte membranes, which was associated with anti-inflammatory effects, providing substantial evidence for this mechanism and the relationship with inflammatory activity [7]. Looking ahead, it is recommended that future studies include a broader analysis of inflammatory markers and pro-resolution molecules using chromatographic methods to corroborate these findings and further elucidate the cellular mechanisms in which the n-6/n-3 PUFA ratio may be involved. Additionally, the use of hypocaloric diets, a primary treatment for obesity, could be explored to improve outcomes by enhancing the modulation of inflammatory pathways and metabolic responses, thus amplifying the anti-inflammatory effects of marine n-3 PUFAs. This combined approach may provide a more comprehensive understanding of dietary interventions in the regulation of inflammation.

These results could provide a guide for future nutritional intervention strategies aimed to improve metabolic health and reduce chronic low-grade inflammation by considering the n-6/n-3 PUFA ratio content as a necessary calculation for a proper diet.

## 5. Conclusions

Regarding diet intervention, the n-6/n-3 PUFA ratio intake improved significantly in both groups, achieving an ideal proportion ≤ 5:1. Moreover, adherence showed no statistically significant differences where the mean percentage in both groups was above 70%.

Therefore, after intervention, improvements in weight, BMI, TG, VLDL-c, and TNFα were observed in both groups; however, in the marine omega-3 group, HDL-c, body fat percentage, and WC improved significantly in men probably due to a greater weight loss compared to the active placebo group. In addition, an improvement in fasting insulin and HOMA index was observed in both groups with no differences between groups.

Moreover, after the intervention, subjects in the marine omega-3 group had a higher reduction in the prevalence of abdominal obesity compared to the active placebo group, and better differences between IL-6, IL-10, MCP-1, and RvD1 changes at the end of the study.

## Figures and Tables

**Table 1:** Nutritional variables of the diet in the study groups.

	Active Placebo Group (n = 19)				Marine Omega-3 Group (n = 21)				
Variables	Baseline	Final	∆	*p* ^1^	Baseline	Final	∆	*p* ^2^	*p* ^3^
Energy (kcal)	2138 (1822–2324)	1616 (1271–1838)	−548.2 (−822.8–−49)	0.038	1928 (1706–3075)	1271 (1046–1713)	−358.41 (−1311–42)	0.060	0.964
Protein (g)	85.5 (58.5–124.7)	82 (73.5–101.5)	−4.5 (−32.2–18.5)	0.754	87 (67.2–109.2)	74.5 (63.7–109.5)	−7.5 (−29.5–15.2)	0.575	0.742
Carbohydrates (%)	48.4 (43.9–52.4)	50.6 (42.9–52.1)	1.3 (−10.1–5)	0.814	48.9 (41.3–54.1)	50.1 (46.9–53.5)	2 (−2.3–6.9)	0.262	0.531
Fat (%)	34.3 (31.5–41)	31.5 (27.5–38.1)	−2.8 (−7.6–7.2)	0.477	35.2 (27.7–38.8)	28 (23.7–30.3)	−6.8 (−9.9–−2.2)	0.028	0.291
SFAs (g)	22 (15.9–32.5)	14.2 (9.1–17.9)	−8.9 (−19–−2.6)	0.034	19.4 (15.2–26.6)	10.2 (8.6–14.6)	−9.7 (−17.6–1.8)	0.047	0.947
MUFAs (g)	30.6 (21.3–38.1)	24.8 (15.6–29.1)	−6.4 (−17–4.4)	0.272	23.6 (15.7–29.5)	14.4 (11–18.5)	−9.3 (−16.4–3.5)	0.114	0.895
PUFAs (g)	15.1 (9.3–21.5)	12.9 (10.6–20.6)	−0.45 (−8.5–6.7)	0.784	11.5 (10.1–14.0)	8.7 (7.2–12.6)	−0.75 (−3.5–1.03)	0.333	0.843
n-3 PUFAs (g)	1 (0.7–1.6)	2.9 (1.4–4.8)	2.0 (0.62–3.31)	0.012	1.2 (0.8–1.9)	1.8 (1.1–4.4)	2.0 (0.03–3.41)	0.023	0.649
n-6 PUFAs (g)	10.9 (6.4–17.4)	8.9 (7.7–11.9)	−1.9 (−10.5–2.9)	0.307	11.4 (8.9–17.1)	6.6 (5.3–7.7)	−3.3 (−5.9–−1.41)	0.012	0.698
n-6/n-3 ratio diet	9.9 (7.5–13.1)	3.4 (2.3–5.2)	−7.0 (−11.0–−3.1)	0.019	9 (7.6–13.3)	2.8 (1.7–5.2)	−5.6 (−10.9–−4.12)	0.003	0.677

Data are represented as median and interquartile range. Kcal: kilocalories, g: grams, n-6/n-3 ratio: grams of n-6 per 1 g of n-3, n-3: omega-3, n-6: omega-6, SFAs: saturated fatty acids, MUFAs: monounsaturated fatty acids, PUFAs: polyunsaturated fatty acids, ∆: final value–baseline value. The Mann–Whitney U test and Wilcoxon test were used. Values of *p* < 0.05 were considered statistically significant. The *p*^1^ value corresponds to the comparison final–baseline on the active placebo group (intragroup). The *p*^2^ value corresponds to the comparison final–baseline on the marine omega-3 group (intragroup). The *p*^3^ value corresponds to the comparison between the groups (intergroup: active placebo vs. omega-3 group).

**Table 2:** Anthropometric and biochemical variables in the study groups.

	Active Placebo Group (n = 19)					Marine Omega-3 Group (n = 21)					
Variables	Baseline	4 Weeks	Final	∆	*p* ^1^	Baseline	4 Weeks	Final	∆	*p* ^2^	*p* ^3^
Anthropometric											
Weight (kg)	103 ± 20	101.5 ± 19.5	100.8 ± 19.2	−2.24 ± 3.4	0.004	96.7 ± 16.1	93.4 ± 15.4	92.3 ± 15.2	−3.76 ± 3.5	0.001	0.157
BMI (kg/m^2^)	36.0 ± 4.7	35.7 ± 5.1	35.5 ± 4.9	−0.50 ± 1.3	0.049	33.6 ± 3.4	32.9 ± 3.1	32.5 ± 3.0	−1.24 ± 1.2	0.001	0.111
Body fat Women (%)	48.2 ± 4.8	47.9 ± 5.5	47.4 ± 5.4	−0.7 ± 1.1	0.110	44.9 ± 3.1	45.5 ± 2.8	44.7 ± 3.7	−0.2 ± 1.8	0.714	0.513
Body fat Men (%)	42.6 ± 5.5	42.3 ± 5.7	40.9 ± 7.4	−1.7 ± 3	0.092	39.5 ± 5.1	38.9 ± 4.8	37.8 ± 5.6	−1.6 ± 1.7	0.015	0.961
WC Men (cm)	117 ±11.4	116.6 ± 11.1	113.7 ± 9.7	−3.2 ± 2.4	0.002	110.8 ± 8.7	107.3 ± 7.3	105.8 ± 7.2	−5.02 ± 3.8	0.001	0.205
WC Women (cm)	98.2 ± 9.5	95.9± 9.5	94.1 ± 9.3	−4.1 ± 2.6	0.003	91.8 ± 3.8	90 ± 5.2	86.8 ± 4.8	−5.0 ± 2.7	0.002	0.531
Biochemical											
TC (mg/mL)	160.4 ± 32	155.1 ± 35.3	160.2 ± 34.9	−1.05 ± 12.4	0.213	171.1 ± 26.6	163.0 ± 23.9	166.4 ± 31.5	−3.63 ± 18.8	0.230	0.629
TG (mg/mL)	167.3 ± 73	139.8 ± 62.1	146.5 ± 65.4	−17.94 ± 47.3	0.019	195.3 ± 77.7	155.5 ± 63.8	155.0 ± 99.5	−42.15 ± 85.3	0.003	0.494
HDL-c Women(mg/mL)	36.3 ± 6.7	35.5 ± 5.1	33.2 ± 5.2	−3.1 ± 4	0.065	39 ± 6.7	37.6 ± 7.3	37.8 ± 7.6	−1.1 ± 4.1	0.465	0.344
HDL-c Men (mg/mL)	34.6 ± 7.6	33.1 ± 8.6	33.6 ± 7.7	−2.2 ± 5.3	0.227	33 ± 5.6	30.8 ± 6.7	29.6 ± 6.6	−3.5 ± 3.8	0.012	0.522
LDL-c (mg/mL)	91.7 ± 24.7	93.1 ± 25.8	97.9 ± 25.2	5.38 ± 16	0.346	97.3 ± 21.0	98.2 ± 21.4	102.2 ± 27	7.15 ± 17.8	0.216	0.754
VLDL-c (mg/mL)	33.5 ± 14.6	27.8 ± 12.5	29.3 ± 13.0	−3.55 ± 9.4	0.014	39.0 ± 15.5	31.1 ± 12.7	30.8 ± 19.9	−8.57 ± 17.0	0.005	0.369
Glucose (mg/mL)	94.0 ± 9.4	95.1 ± 7.2	98.3 ± 9.6	4.27 ± 9.27	0.154	96.4 ± 10.8	95.0 ± 9.1	97.8 ± 10.7	1.89 ± 13.3	0.272	0.534

Data are presented as mean ± standard deviation, analyzed by repeated measures ANOVA (adjusted by biological sex). BMI: body mass index, WC: waist circumference, TC: total cholesterol, TG: triglycerides, HDL-c: high-density lipoprotein cholesterol, LDL-c: low-density lipoprotein cholesterol, VLDL-c: very low-density lipoprotein cholesterol. ∆: Final mean value–baseline mean value. The intragroup comparisons (baseline vs. final) were performed with the paired Student’s *t*-test. Delta (∆) comparisons between groups (intergroup) were analyzed with the unpaired Student’s *t*-test. Values of *p* < 0.05 were considered statistically significant. The *p*^1^ value corresponds to the comparison final–baseline on the active placebo group (intragroup). The *p*^2^ value corresponds to the comparison final–baseline on the marine omega-3 group (intragroup). The *p*^3^ value corresponds to the comparison between the groups (intergroup: active placebo vs. omega-3 group).

**Table 3:** Prevalence of abdominal obesity in the study groups.

Group	Baseline (%)	Final (%)	*p* Value
Active placebo (n = 19)	88.9	83.3	0.978
Marine omega-3 (n = 21)	95	60	0.016

Data are presented as percentages. McNemar test, *p* < 0.05, was considered statistically significant. Abdominal obesity was defined as waist circumference ≥ 88 cm in women and ≥102 cm in men according to ATP-III [12].

**Table 4:** Inflammatory variables in the study groups.

	Active Placebo Group (n = 19)				Marine Omega-3 Group (n = 21)				
Variables	Baseline	Final	∆	*p* ^1^	Baseline	Final	∆	*p* ^2^	*p* ^3^
Inflammmatory									
TNF-α (pg/mL)	0.52 (0.4–0.8)	0.07 (0.05–0.11)	−1.85 (−4.52–−0.71)	0.002	0.61 (0.4–1.6)	0.07 (0.04–0.17)	−1.82 (−5.74–−0.59)	0.001	0.745
IL-6 (pg/mL)	2.9 (0.9–7.8)	4.7 (0.6–7.7)	0.03 (−0.59–1.84)	0.407	2.3 (0.7–6.4)	2.0 (0.3–4.4)	−0.67 (−2.72–−0.01)	0.010	0.015
hs-CRP (mg/L)	3.4 (2.7–5.7)	2.7 (2.7–6.6)	−0.7 (−1.30–0.30)	0.296	4.5 (2.0–10.1)	2.4 (1.5–8.2)	−0.5 (−3.07–0.87)	0.271	0.603
IL-10 (pg/mL)	5.9 (3.8–8.0)	4.58 (3.3–5.1)	−2.0 (−5.0–0.05)	0.081	5.3 (4.5–7.2)	7.02 (4.3–12.1)	1.4 (−0.7–4.6)	0.035	0.001
MCP-1 (pg/mL)	233 (204–337)	277 (234–313)	18.3 (−97.3–66.35)	0.758	266 (215–295)	235 (174–274)	−29.6 (−94.9–5.50)	0.040	0.064
RvD1 (pg/mL)	443.3 (358–545)	403 (264–567)	−16.8 (−237.8–92.50)	0.586	466 (392–563)	562 (473–779)	129.3 (−90.1–193.5)	0.048	0.041

Data are presented as median and interquartile range. TNF-α: tumor necrosis factor alpha, IL-6: Interleukin 6, hs-CRP: high-sensitivity C-reactive protein, IL-10: Interleukin 10, MCP-1: monocyte chemoattractant protein 1, RvD1: Resolvin D1, ∆: final mean value–baseline mean value. Values of *p* < 0.05 were considered statistically significant. The Mann–Whitney U test and Wilcoxon test were used. The *p*^1^ value corresponds to the comparison final–baseline on the active placebo group (intragroup). The *p*^2^ value corresponds to the comparison final–baseline on the marine omega-3 group (intragroup). The *p*^3^ value corresponds to the comparison between the groups (intergroup: active placebo vs. omega-3 group).

## Data Availability

The data presented in this study are available upon request from the corresponding author.

## Supplementary Materials

The following supporting information can be downloaded at: https://www.mdpi.com/article/10.3390/healthcare13020103/s1, Supplementary Table S1: Comparison of baseline nutritional variables in the study groups.

## Abbreviations

ALA	Alpha-linolenic acid
ANOVA	Repeated measures analysis of variance
BF%	Body fat percentage
BMI	Body mass index
DHA	Docosahexaenoic acid
EPA	Eicosapentaenoic acid
FFAR4	Free fatty acid receptor 4
GPC	Guías de práctica clínica
HDL-c	High-density lipoprotein cholesterol
HOMA-IR	Homeostasis model assessment
IFN-γ	Interferon gamma
IL-10	Interleukin-10
IL-6	Interleukin-6
IQR	Interquartile range
LDL-c	Low-density lipoprotein cholesterol
MaR	Maresins
MCP-1	Monocyte chemoattractant protein-1
n-3	Omega-3 fatty acids
n-6	Omega-6 fatty acids
NF-κB	Nuclear factor κB
NOM-037	Norma oficial Mexicana 037
NOM-043	Norma oficial Mexicana 043
OB	Obesity
PD	Protectins
PUFAs	Polyunsaturated fatty acids
Rv	Resolvin
RvD1	Resolvin D1
SD	Standard deviation
SPMs	Pro-resolving mediators
TC	Total cholesterol
TG	Triglycerides
TGF-β	Transforming growth factor beta
TNF-α	Tumor necrosis factor α
VLDL-c	Very low-density lipoprotein cholesterol
WC	Waist circumference
