# Supplementary material for: Effect of a Diet Supplemented with Marine Omega-3 Fatty Acids on Inflammatory Markers in Subjects with Obesity: A Randomized Active Placebo-Controlled Trial
Source: Healthcare (Basel). 2025 Jan 8;13(2):103. doi: 10.3390/healthcare13020103 (PMC11764561; doi:10.3390/healthcare13020103)
Supplement: Supplementary file 1 [file healthcare-13-00103-s001.zip › healthcare-3327724-supplementary.pdf]

Supplementary Table S1. Comparison of baseline nutritional variables in the study groups.

Baseline intake			
**Variables**	*Active placebo group (n=19)*	*Marine omega-3 group (n=21)*	*p*
**Energy (kcal)**	2138 (1822–2324)	1928 (1706-3075)	0.976
**Protein (g)**	85.5 (58.5-124.7)	87 (67.2-109.2)	0.974
**Carbohydrates (%)**	48.4 (43.9-52.4)	48.9 (41.3-54.1)	0.869
**Fat (%)**	34.3 (31.5-41)	35.2 (27.7-38.8)	0.692
**SFAs (g)**	22 (15.9-32.5)	19.4 (15.2-26.6)	0.489
**MUFAs (g)**	30.6 (21.3-38.1)	23.6 (15.7-29.5)	0.138
**PUFAs (g)**	15.1 (9.3-21.5)	11.5 (10.1-14.0)	0.291
**n-3 PUFAs (g)**	1 (0.7–1.6)	1.2 (0.8–1.9)	0.527
**n-6 PUFAs (g)**	10.9 (6.4–17.4)	11.4 (8.9–17.1)	0.684
**n-6:n-3 ratio**	9.9 (7.5–13.1)	9 (7.6–13.3)	0.927
**20:5n-3 EPA (g)**	0.006 (0.002-0.017)	0.004 (0.001-0.022)	0.749
**22:6n-3 DHA (g)**	0.051 (0.008-0.060)	0.058 (0.004-0.089)	0.988
**22:5n-3 DPA (g)**	0.016 (0.007-0.022)	0.013 (0.004-0.031)	0.819
**18:3n-3 ALA (g)**	1.058 (0.716-2.269)	1.230 (0.860-1.66)	0.820
**20:4n-6 AA (g)**	0.187 (0.075–0.267)	0.210 (0.067-0.321)	0.584
**18:2n-6 LA (g)**	11.80 (7.468–17.91)	11.73 (9.223-17.310)	0.682

Mann-Whitney U test. Data is represented as median and interquartile range. Values of $p < 0.05$ were considered statistically significant. Kcal: Kilocalories, g: grams, n-6: n-3 ratio: grams of omega-6 per 1 gram of omega-3, n-3: omega 3, n-6: omega 6, SFAs: Saturated Fatty Acids, MUFAs: Monounsaturated Fatty Acids PUFAs: polyunsaturated fatty acids, EPA: Eicosapentaenoic acid, DHA: Docosahexaenoic acid, DPA: Docosapentaenoic acid, ALA: Alpha-linolenic acid, AA: Arachidonic acid, LA: Linoleic acid.
